# Supplementary figures and images for: The Value of Ultrasonic Elastography in Detecting Placental Stiffness for the Diagnosis of Preeclampsia: A Meta-Analysis
Source: Diagnostics (Basel). 2023 Sep 9;13(18):2894. doi: 10.3390/diagnostics13182894 (PMC10527587; doi:10.3390/diagnostics13182894)

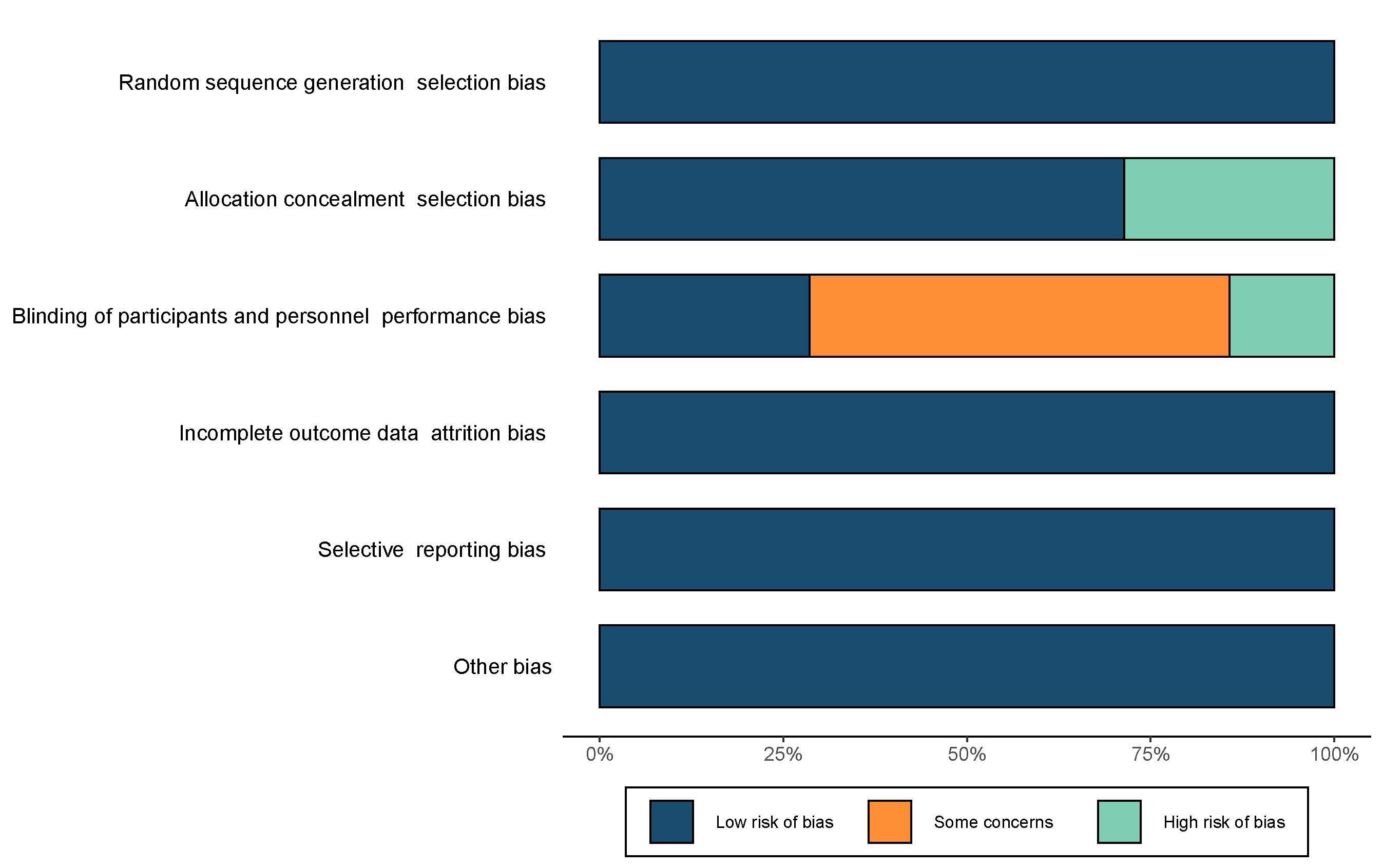

Supplement: Supplementary file 1 [file diagnostics-13-02894-s001.zip › diagnostics-2599854-supplementary.tif]
